# Surgical Excision of a Pilonidal Sinus at the Neck Region

**DOI:** 10.7759/cureus.13984

**Published:** 2021-03-18

**Authors:** Zaher Mikwar, Saeed N Albukhari, Sultan A Neazy, Hossein A Filimban

**Affiliations:** 1 Surgical Oncology, King Abdulaziz Medical City, Ministry of National Guard Health Affairs, Jeddah, SAU; 2 College of Medicine, King Saud Bin Abdulaziz University for Health Sciences College of Medicine, Jeddah, SAU

**Keywords:** pilonidal sinus surgery, sinus excision, neck reconstruction, pilonidal sinus disease, surgery, management, abscess

## Abstract

Pilonidal sinus (PNS) is a clinically diagnosed condition caused by hair entrapment underneath the skin leading to a granulomatous reaction and the formation of a tract. PNS is commonly found in the sacrococcygeal area (natal cleft). However, this is a rare case presentation of a male patient with PNS located at the back of his neck region as there were only four similar conditions reported in the literature review. The patient was first managed medically as a case of hidradenitis suppurativa by the dermatology department. Later on, when the treatment failed, he was treated surgically as a case of PNS with an excellent outcome. The patient was seen at the outpatient clinic one week after surgery. The wound was healed completely and totally recovered.

## Introduction

Pilonidal sinus (PNS) is a condition where the area underneath the skin is infected as a result of hair follicle entrapment leading to a granulomatous reaction and the formation of a tract that extends along the surface of the skin. R. M. Hodges first mentioned the term “Pilonidal” in 1880, thus, coining the term. However, the earliest description of the disease was done by Herbert Mayo in 1830 [1]. Later on, it was also known as “Jeep disease” as it affected many soldiers who drove jeeps on rough surfaces during the world war [1]. PNS has a male predominance with a ratio of 2.2:1 mainly affecting young adults and middle-aged people; moreover, 26 per 100,000 is the estimated incidence of the disease [2]. Multiple risk factors were identified: personal hygiene, obesity, prolonged driving, hirsuteness, male sex, family history, and sedentary occupation. The most common location of PNS is the sacrococcygeal area (natal cleft) but can also less likely occur in other parts of the body such as the umbilicus, axillae, and finger webs. The clinical presentation of PNS is varied in which it can be asymptomatic or exhibiting inflammatory processes such as tenderness, erythema, hotness, discharge, and sometimes abscess formation [3]. Although PNS is clinically diagnosed with no further tests required, imaging modalities might help confirm the diagnosis when the diagnosis is less clear and assess the severity of the disease [2]. PNS treatment is categorized into operative, with either open or closed wound methods depending on how clear was the sinus at the time of surgery, or non-operative treatment and sometimes the combination of the two. Both are considered effective when used in the appropriate setting. The treatment approach is tailored depending on the patient’s preference and the severity of the disease [2]. Up to our knowledge, there have been only four similar cases reported of the neck-located PNS in the literature review [4]. Therefore, our aim is to present a rare case of a male patient who developed a neck PNS and was successfully managed operatively.

## Case presentation

**Figure 1:**
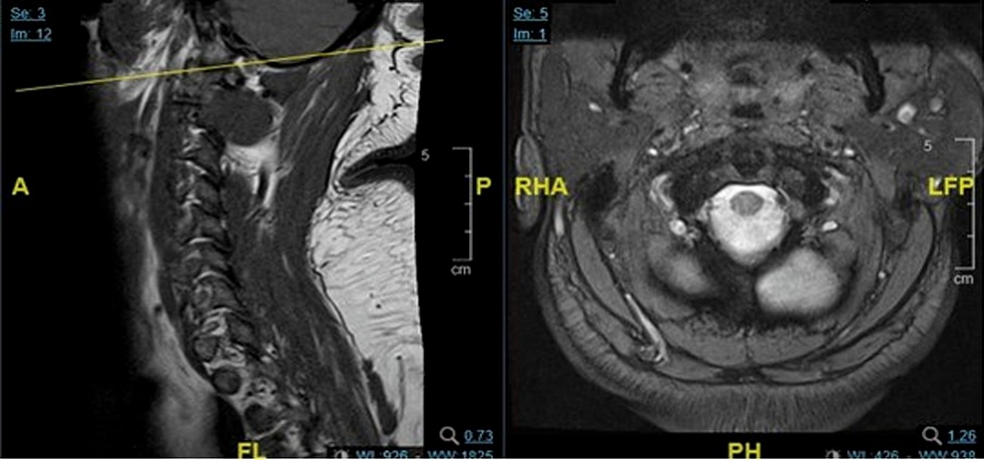
Magnetic resonance imaging (MRI) with no significant findings. A = anterior, P = posterior, FL= feet left, PH = posterior head, RHA = right head anterior, LFP= left feet posterior

A 43-year-old male presented to the dermatology out-patients department complaining of multiple infected sebaceous cysts located at the upper nape of the neck for one year prior to presentation. The patient reported that the cysts were painful, red and with minimal to moderate discharge. Moreover, he has a positive family history of psoriasis. Upon clinical examination, the patient has had a body mass index (BMI) of 31.87 kg/m^2^. Also, he had tenderness, erythema, moist, and tightened hairy skin around the infected sebaceous cysts. There was massive hyperpigmented seborrheic keratosis around the abdomen, chest, and back. In addition, hair loss was noted in the lower limbs. The patient was diagnosed by the dermatology department as hidradenitis suppurativa and managed with the following medications: Mupirocin 2% ointment, potassium permanganate 0.01% solution, and doxycycline capsules for 15 days. After the failure of the treatment course, he was referred to the surgical clinic. While inspecting the sinus tract, there were multiple small holes that mimic the appearance of the natal cleft PNS. Lab investigations including serology testing for autoimmune diseases (anti-SM, anti-centromere, antinuclear antibody, and anti-SCL70) were all normal. Magnetic resonance imaging (MRI) was normal and there were no significant findings (Figure 1).

**Figure 2:**
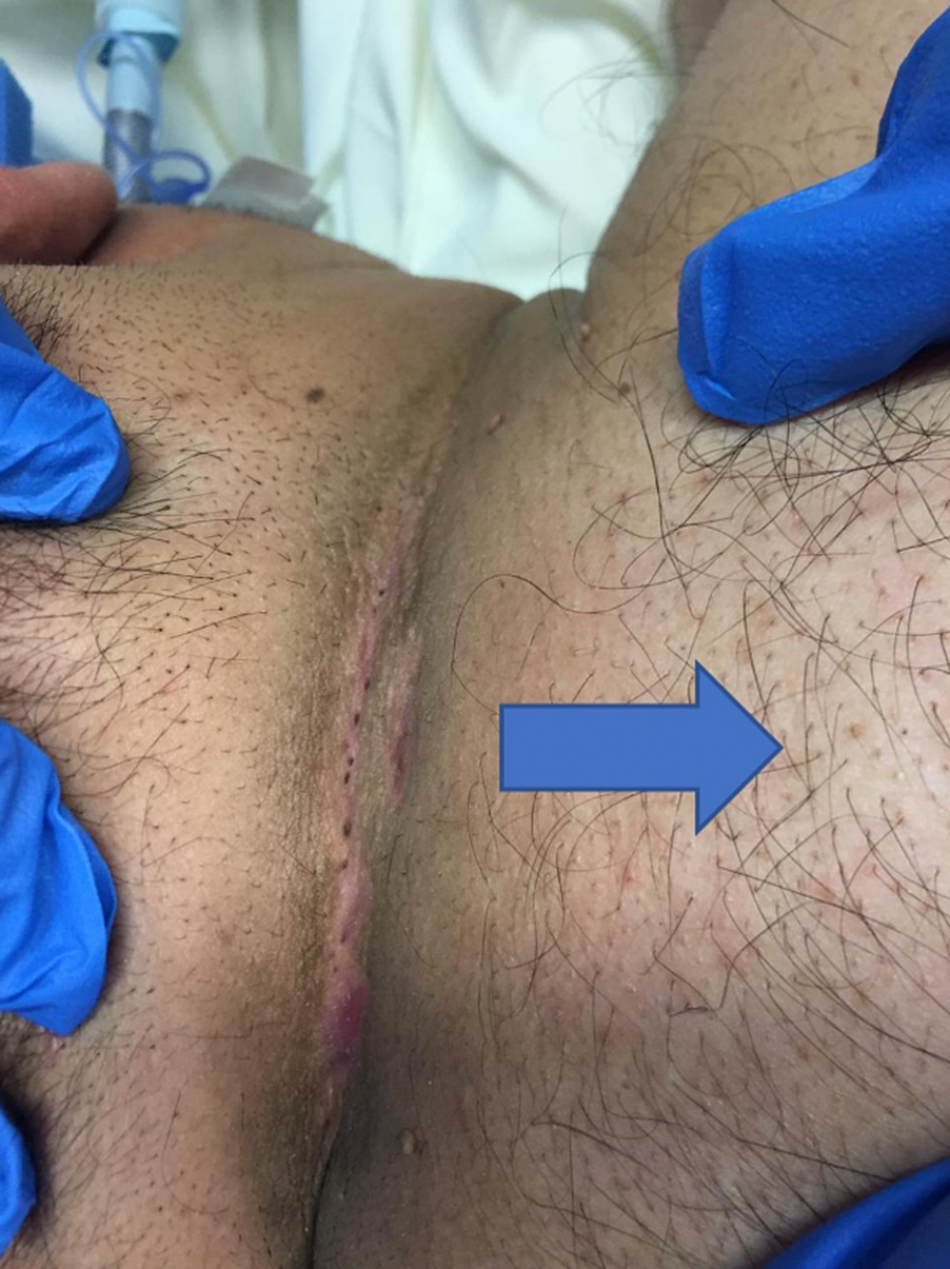
Hairy neck containing the pilonidal sinus (the arrow pointing to the trunk area of the patient).

**Figure 3:**
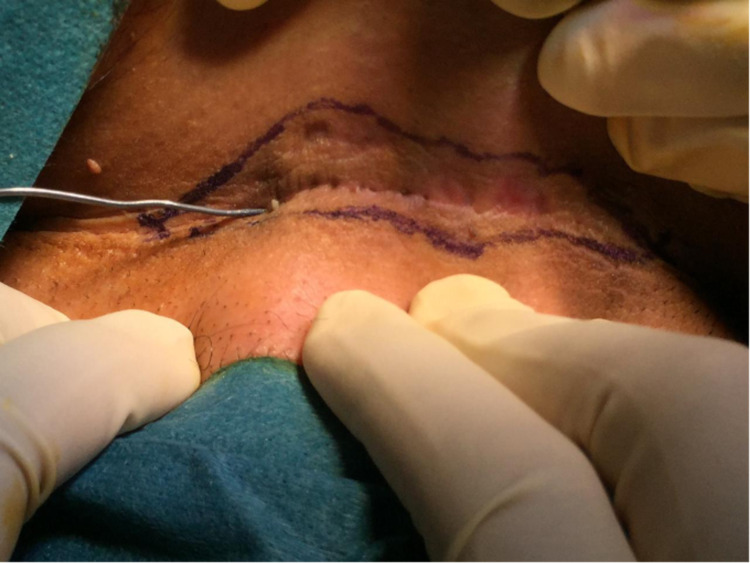
The incision line was marked, the sinus was propped and methylene blue was injected.

**Figure 4:**
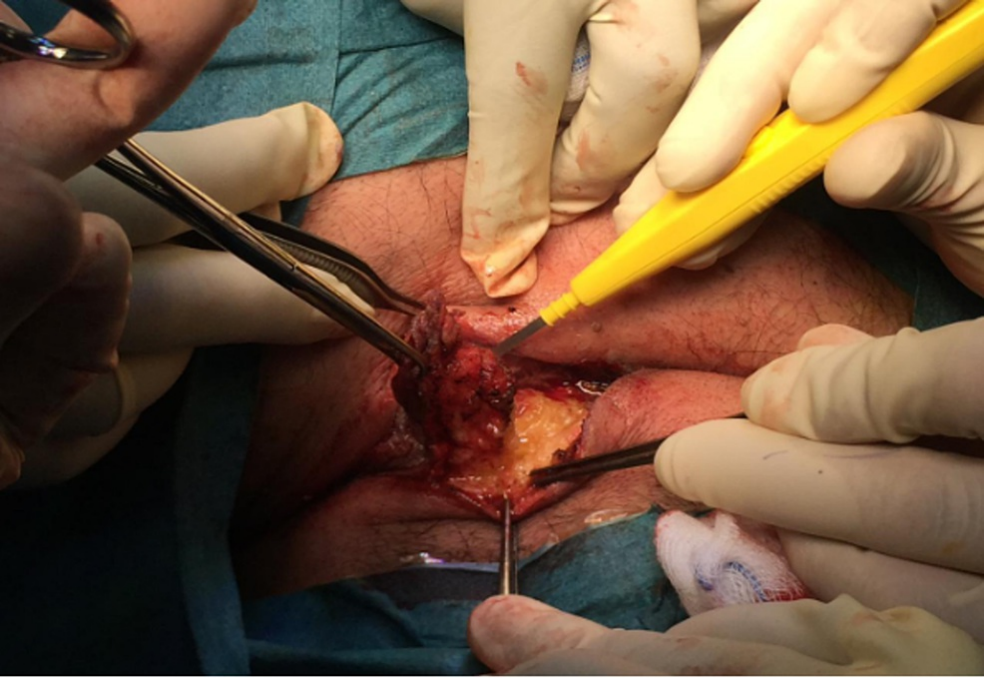
Dissecting the tract using electrical cautery.

**Figure 5:**
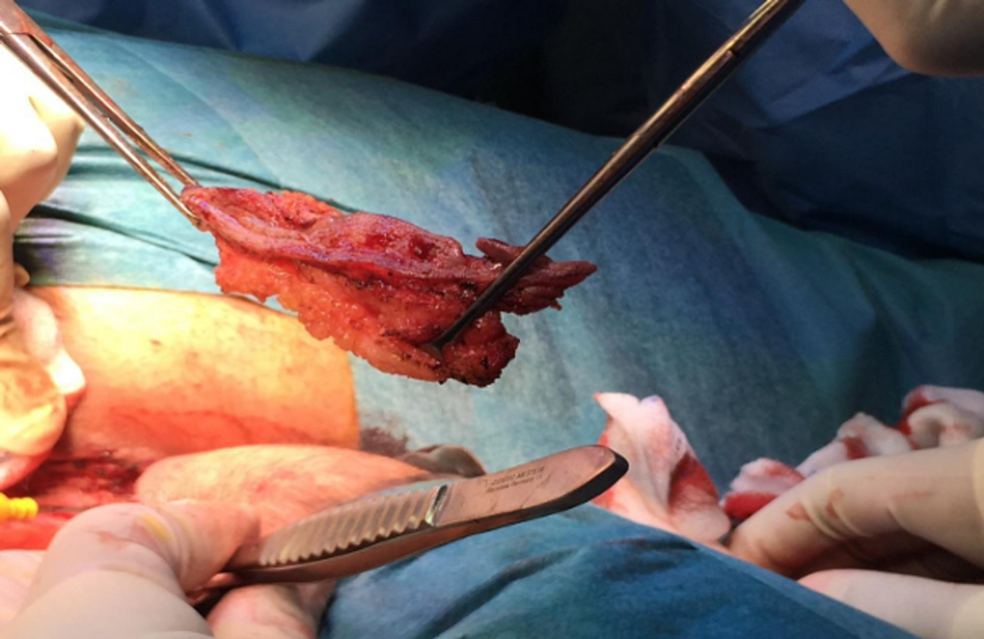
The pilonidal sinus tract after excision.

**Figure 6:**
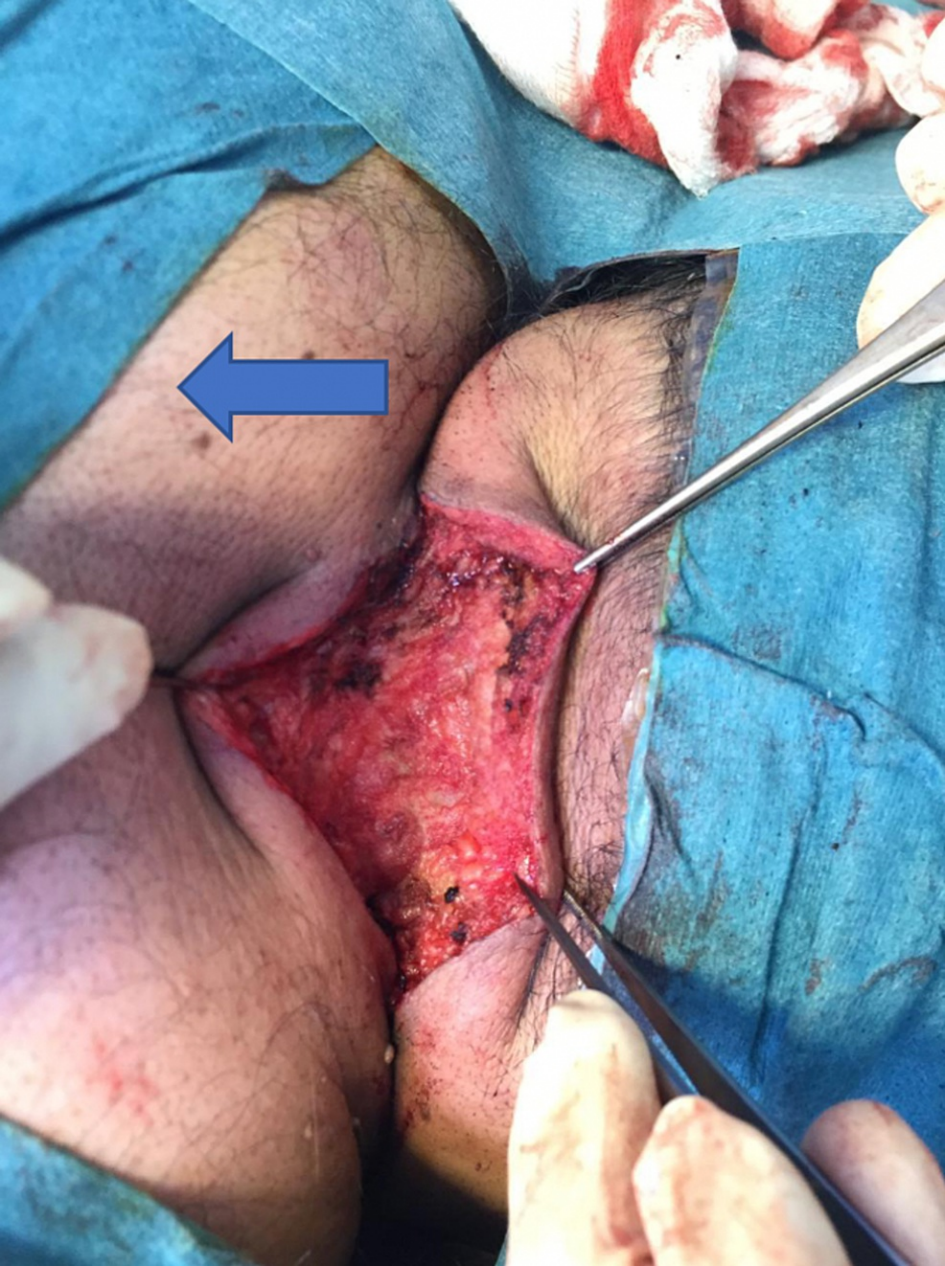
Healthy tissues after excising the sinus (the arrow pointing to the trunk area of the patient).

During surgery, 3 ml of methylene blue mixed with normal saline was injected through one of the small holes to make sure there are no other side-tracks inside the sinus. Using a marker pen, the line of incision was made to include all the small holes. After that, the surgical incision was created and the sinus was excised completely by using electrocauterization (Figures 2-6).

 

 

**Figure 7:**
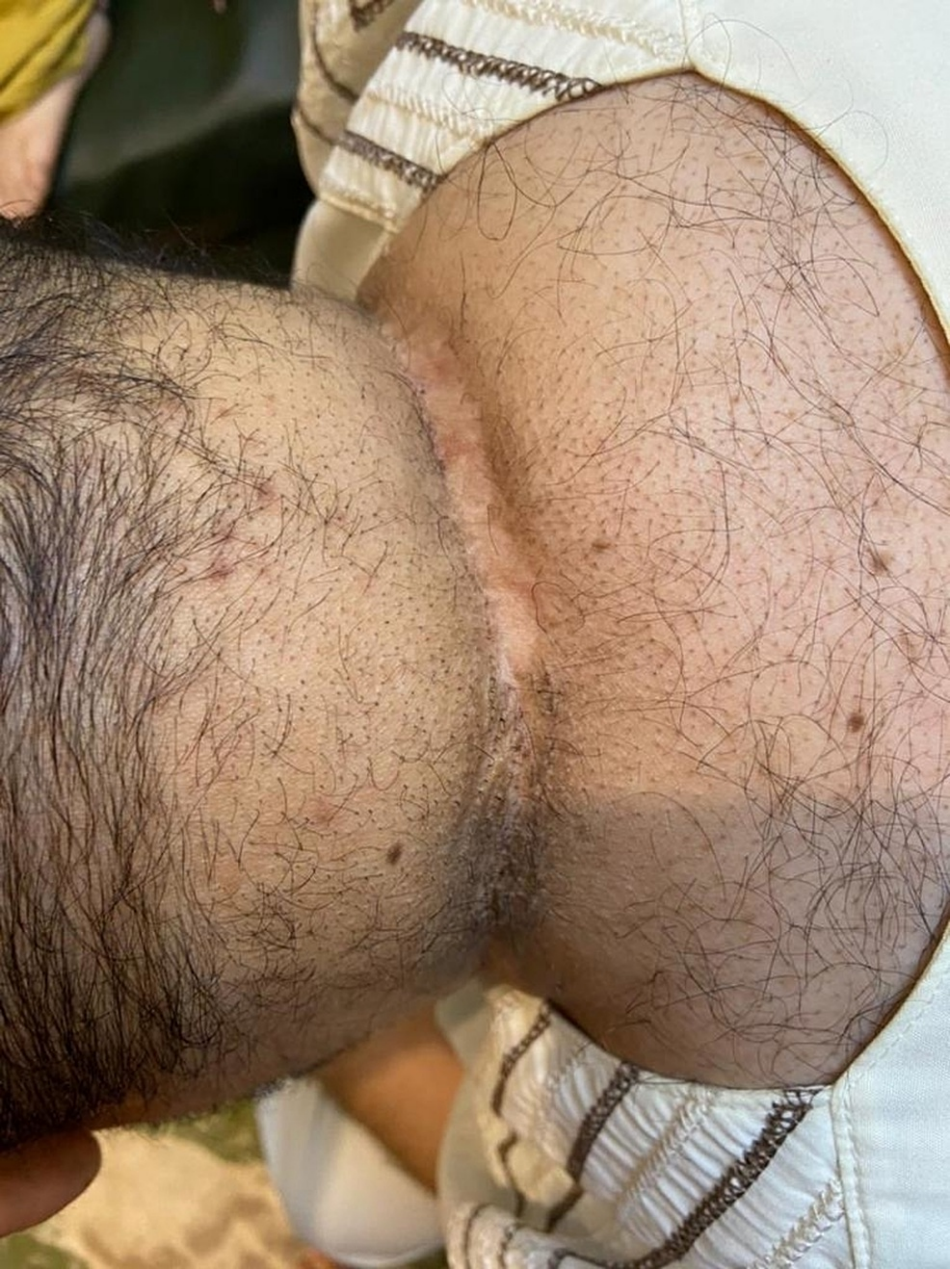
Three years after surgery, nicely healed wound; no recurrence.

The patient tolerated the procedure well without any complications and was shifted to the recovery room in a good condition. The excised sinus was sent for histopathological evaluation. The report states that the specimen received consisted of elongated fragments of hairy skin and underlying fatty tissue 7 x 1.5 x 1 cm in diameter. Both pre-and post-operative diagnoses were stated as PNSs. During the follow-up, the patient's wound healed completely and totally recovered (Figure 7). 

## Discussion

The prevalence of PNS in Saudi Arabia is not known; however, it is not considered a rare disease [3]. Being male seems to be a major risk factor for developing PNS in Saudi Arabia as multiple studies revealed that male patients are four times more prone than females [3,5]. This finding could be explained by the fact that men are more associated with other risk factors than females such as prolonged driving and a hairy body type. Moreover, the climate could be a risk factor as hot and humid areas like the middle east, for example, had a higher prevalence of PNS [5].

PNS is usually diagnosed clinically through history and physical examination. However, an imaging modality such as ultrasound or MRI could be used to aid the diagnosis, especially when the PNS presents at odd locations other than the sacrococcygeal area, and to rule out other differential diagnoses which may include hidradenitis suppurativa, abscesses, inflammatory bowel disease, or fistula. Furthermore, skin cancer is also considered one of the dangerous differential diagnoses. If the treatment approach was surgical intervention, the excised specimen of PNS is recommended to be sent for histopathology examination to be sure that it is a benign PNS as a study conducted by Yuksel et al., in which they reviewed articles that are concerned with PNS and histological evaluation of the surgical specimen from 2000 to 2018, found that 83 patients had malignancy with PNS from 22 articles [6]. Taking into consideration that these findings are rare, it is essential to send the resected PNS for histopathological assessment [6].

PNS is treated using operative techniques, non-operative techniques, or both. Multiple factors should be taken into consideration when choosing one treatment option over the other which are the recurrence rate, post-operative pain, wound care and healing, hospital stay, cost-effectiveness, and early full physical activity attainment [1,5]. Furthermore, the extent and severity of the disease could outline the treatment approach. For example, when PNS presents with an abscess, the preferred treatment would be incision and drainage [2]. The non-operative treatment methods that could be used as a primary treatment or as an adjunct include depilation, phenol, and fibrin glue. The reported benefits were decreased recurrence rate and early resumption of daily activities [2]. Others could only be used as an adjunct. For instance, platelet-rich plasma and hyperbaric oxygen had evidence of decreased wound healing time [2]. As for the operative techniques, there are many options that range from minimally invasive approaches such as pit-packing to more complicated ones such as surgical excision. Due to the minor trauma caused by these minimally invasive techniques, the patients can get back to work earlier making this outcome a big advantage [7]. One of the new minimally invasive operations is endoscopic PNS treatment [2]. Moreover, the video-assisted technique showed a great result of achieving a healing rate of 95%, being safe, and earning good patient satisfaction [2]. On the other hand, surgical excisional techniques could be divided into an open method or a closed method. In the open method, the wound is left open to heal by secondary intention, and this type of approach is considered the most popular one because of its simplicity and its low recurrence rate. However, the quality of life of the patients could be affected severely due to the prolonged healing time of the wound; therefore, the open method is preferred when the lesions are small as they heal faster in comparison with a large wound that requires much more time to heal [5,7]. For the closed method, the wound is closed by a suture either in the midline or off-midline which facilitates wound healing and a quicker return to work [7]. It is worth noting that the midline suture had a higher recurrence rate and wound complication compared to both the open method and off-midline suture, which made this technique not recommended [5,7]. The off-midline techniques usually utilize a flap to place the post-operative wound laterally. There are various types of off-midline techniques and the most famous ones include the Karydakis flap, Bascom flap, and the Limberg flap. Moreover, the off-midline technique had a better result when compared to the open method in terms of recurrence rate based on the current evidence [7]. Although there are numerous research articles regarding PNS and its treatment, the optimal treatment is yet to be agreed upon.

Miyata et al. reported the first case of neck PNS in 1992 [8]. Their patient was a 21-year-old obese male with a BMI of 33 kg/m^2^ and a history of treated sacral PNS. He presented with a left nuchal abscess 7 cm in diameter. He had this problem four years prior and underwent incision and drainage five times in three different health care centers without reaching the proper diagnosis. The abscess still recurred along with suppurative discharge. The patient achieved complete remission after total excision of the lesion and the histopathological evaluation showed characteristics of PNS. The second case of neck PNS was reported by Meher et al. in 2005 [9]. The patient was 24-year-old and was complaining of multiple sinuses on the right side of the upper neck. These sinuses were chronically discharging and had been present for the last three years. The patient reported a shaving trauma in the neck prior to the development of the sinuses. He underwent surgical excision of the sinuses with primary repair after the injection of methylene blue and had a complete recovery. The histopathological examination showed that the lesion had features of PNS. The third case of neck PNS was about a 20-year-old female with posterior neck sinuses lasting for one year [10]. Moreover, these sinuses were accompanied by a yellowish discharge and skin erythema. Excision of the sinus was performed and was sent for histopathological evaluation. The diagnosis was made as PNS in the neck region. Tomas et al. presented the last case of neck PNS [4]. The case was about a 37-year-old male who presented with intermittent pain and lateral neck discharge for several months. At first, he was given antibiotics then excision of the sinus was done. In the histopathological assessment, PNS with hair tuft and necrotic debris was found. In the reported cases as well as in our case, patients complained of discharge from the neck [4,8-10]. This might seem to be a distinct feature of neck PNS. Therefore, atypical PNS is recommended to be considered one of the differential diagnosis whenever patients complain of neck discharge. Total excision of the sinuses and primary closure also in these cases proved to be curative with no complication [4,8-10].

## Conclusions

In summary, this case was representing a male patient with a PNS located in the neck region. This site is considered rare compared to other most common sites such as the sacrococcygeal area. In the beginning, the patient was managed medically by the dermatology department as a case of hidradenitis suppurativa using ointments and antibiotics. Due to treatment failure, the patient was referred to the surgical department and underwent surgical excision of the sinus. Surgical management has provided good and very satisfying results for our patient. Upon one-week follow-up, the wound has healed completely without any complication.
